# Towards a Systems Approach in the Genetic Analysis of Archaea: Accelerating Mutant Construction and Phenotypic Analysis in *Haloferax volcanii*

**DOI:** 10.1155/2010/426239

**Published:** 2010-12-23

**Authors:** Ian K. Blaby, Gabriela Phillips, Crysten E. Blaby-Haas, Kevin S. Gulig, Basma El Yacoubi, Valérie de Crécy-Lagard

**Affiliations:** Department of Microbiology and Cell Science, University of Florida, Gainesville, FL 32611-0700, USA

## Abstract

With the availability of a genome sequence and increasingly sophisticated genetic tools, *Haloferax volcanii* is becoming a model for both Archaea and halophiles. In order for *H. volcanii* to reach a status equivalent to *Escherichia coli, Bacillus subtilis*, or *Saccharomyces cerevisiae*, a gene knockout collection needs to be constructed in order to identify the archaeal essential gene set and enable systematic phenotype screens. A streamlined gene-deletion protocol adapted for potential automation was implemented and used to generate 22 *H. volcanii* deletion strains and identify several potentially essential genes. These gene deletion mutants, generated in this and previous studies, were then analyzed in a high-throughput fashion to measure growth rates in different media and temperature conditions. We conclude that these high-throughput methods are suitable for a rapid investigation of an *H. volcanii* mutant library and suggest that they should form the basis of a larger genome-wide experiment.

## 1. Introduction

Since the identification of the three-kingdom paradigm over 30 years ago [1], the quest for model archaeal organisms, representing each of the main groups, has been ongoing. To date, of the culturable Archaea, *Sulfolobus* sp. remain amongst the most intensely investigated and have become the subject of modern postgenomics experimental techniques [2, 3], yet *in vivo* studies in these organisms have lagged, as robust genetic methodologies are lacking (although this is now being addressed [4, 5]). The ability of thermophiles, such as *Thermococcus kodakaraensis* and *Pyroccocus furiosus*, to thrive at in excess of 95°C is of great interest (particularly to industrials). However, whilst *T. kodakaraensis* has a well-developed genetic system, optimal growth at high temperatures coupled with anaerobic growth requirements puts these organisms out of reach of nonspecialist laboratories [6–8]. The methanogens received an early lead with the first available archaeal genome sequence, *Methanococcus jannaschii* [9], and genetic manipulation techniques have been described in the closely related *Methanococcus maripaludis* [10, 11]. Yet, as strict anaerobes, their manipulation is also still restricted to specialists. 

Thanks to the endeavours of a growing community of investigators (see http://www.haloferax.org/), *Haloferax volcanii* is filling the role of model archeaon and model halophile. Its relatively short generation time of <4 hours [12, 13], ease of growth under laboratory conditions, genome-sequence availability [14], and development of genetic tools [15–17] (including shuttle, expression and suicide vectors using auxotrophy, and antibiotic-based selections) allow gene deletions to be achieved with relative ease. This combination of feats has enabled rapid progress in the study of halophiles and has been aided by the implementation of microarray and proteomics experiments in the closely related organism *Halobacterium salinarum* [18–21]. Hence, the *H. salinarum*/*H. volcanii* pair can be considered to be the most advanced model organisms in Archaea.

The construction of genome-wide gene deletion libraries have been one of the major achievements of the postgenomic era. Initially, such collections were generated using random mutagenesis approaches, and strain-sets using transposon insertions have been described for *Escherichia coli* [22], *Corynebacterium glutamicum* [23], *Haemophilus influenza* [24], and *Pseudomonas aeruginosa* [25, 26] among others. Yet, the potential polar effects of the insertion on downstream genes and the lack of certainty of gene-inactivation make transposon libraries far from ideal. For these reasons, targeted gene-deletion libraries have become the method of choice, despite higher cost and labor requirements. Such mutant collections have been constructed in multiple model organisms [27–29] and have greatly aided gene-function studies [30–32]. The genetic tools now available for halophiles, together with the logistical knowledge gained from previous genome-wide gene deletion libraries, is enabling a systematic analysis to be applied for the first time in an archaeal species. Systematic, and potentially automated, analysis of gene-deletion collections will enable rapid identification of strain phenotypes and should aid the annotation of hypothetical proteins.

As a proof-of-principle case study towards this goal, and to identify suitable approaches for the study of such a library, we analyzed 22 gene-deletion mutations generated in our laboratory using easily scalable high-throughput methods. Here, we report the statistics on the success of this endeavour and present the techniques used to establish accelerated genetics in *H. volcanii*. Furthermore, we employed high-throughput array-based phenotyping approaches, with the potential for scalability and automation, to aid the study of these and other mutants and demonstrate that *H. volcanii* is perfectly amenable to these techniques (assuming the predicted 4209 genes, the mutants analyzed here represent approximately 0.6% of all possible gene deletions). We propose the methods used here form the basis of a high-throughput phenotyping analysis of *H. volcanii*.

## 2. Materials and Methods

### 2.1. Strains, Plasmids, Media, and Transformation Procedures

All strains and plasmids used in this study are detailed in Supplementary Tables 1 and 2 available at doi:10.1155/2010/426239. *H. volcanii* H26 was used as the parent strain. *E. coli* was routinely grown in LB-Lennox (LB) (Fisher) or LB agar (Fisher) at 37°C, supplemented when required with ampicillin (Amp; 100 *μ*g mL^−1^), isopropyl *β*-D-1-thiogalactopyranoside (IPTG; 0.2 mM) and bromo-chloro-indolyl-galactopyranoside (X-gal; 40 *μ*g mL^−1^). When required, novobiocin was added to a final concentration of 0.3 *μ*g mL^−1^. *H. volcanii* cells were routinely grown at 44°C (unless specified) in Hv-YPC, HvCA, or CDM. *H. volcanii* media was made according to the recipes provided in the HaloHandbook [12]. Transformations of chemically competent *E. coli* were performed as described by the manufacturer's directions (Invitrogen, CA). Transformation of *H. volcanii* was performed as described in the HaloHandbook using the “standard PEG-mediated transformation of Haloarchaea” protocol. Briefly, spheroplasts were made by adding 100 *μ*l 0.5 M EDTA (pH 8.0) to 1 mL concentrated cells and incubated at room temperature for 10 min. 2 *μ*g of plasmid DNA were added to 100 *μ*l spheroplasts and incubated for 5 min. 100 *μ*l 60% (v/v) PEG_600_ was added and mixed gently. The mixture was incubated at room temperature for 30 min. Cells were recovered by adding recovery solution, as described in the HaloHandbook, and incubated for 4 hrs at 44°C before plating onto the appropriate medium.

### 2.2. Gene Deletion Strain Construction

**Figure 1:**
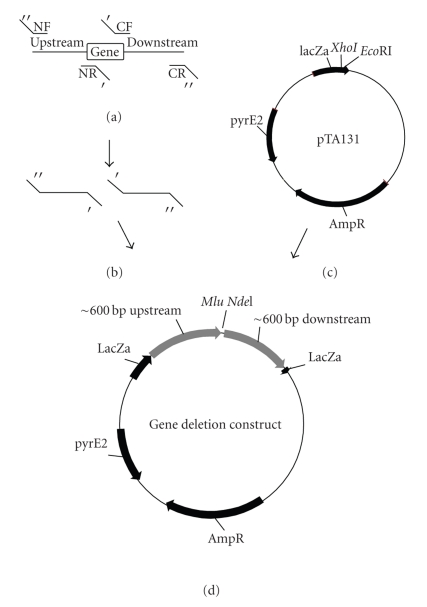
Generation of *H. volcanii* gene deletion constructs. Regions of approximately 600 bp flanking the gene of interest were amplified from the *H. volcanii* chromosome (a) Regions of complementarity between primers are ^'^ and ^”^. Complementarity sequences were 15 b, as follows: NF: CGGGCCCCCCCTCGAG; NR: GACGCGTTCATATGC; CF: GCATATGAACGCGTC CR: CGGGCTGCAGGAATTC and were designed to yield unique *Mlu*I *Nde*I sites. Plasmid pTA131 was digested with *EcoR*I and *Xho*I (c) and the three fragments recombined using In-Fusion (d). Refer to Materials and Methods for details of the techniques used.

Plasmids to delete *H. volcanii* genes were constructed as follows. Regions of approximately 600 bp upstream and downstream of the gene to be deleted were amplified by polymerase chain reaction (PCR) using Phusion Hot Start polymerase (Finnzymes, Espoo, Finland) from purified *H. volcanii* genomic DNA (prepared as described in [17]) with the primers listed in Supplemental Table 3. Primers were designed such that they contained 18–20 bases of homology to the chromosomal DNA. The reverse primer for the upstream fragment and the forward primer for the downstream fragment also contained a linker of 15 bases that enabled joining of the two fragments by recombination (described below). The linker was designed to incorporate a novel 15 bp spacer between the up- and downstream fragments containing unique *Nde*I and *Mlu*I cut sites (Figure 1). The forward primer for the upstream fragment and the reverse primer for the downstream fragment also contained 15 bases of homology to the pTA131 (Figure 1). pTA131 was linearized by cutting with *Xho*I and *Eco*RI. After the two fragments and linearized vector were gel-purified (QiaQuick, Qiagen), they were combined in a 5 : 5 : 1 (upstream fragment:downstream fragment:linearized pTA131; see Figure 1) molar ratio (corrected to include 100 ng linear vector) and recombined using In-Fusion as directed by the manufacturer (Clontech, CA). Reactions were incubated for 15 min at 37°C followed by 15 min at 50°C. Subsequent to incubation, 0.2 *μ*l was transformed into Top10 cells (Invitrogen, CA) according to the manufacturer's directions and the cells plated onto LB plus Amp, IPTG, and X-gal. Clones were screened by PCR using M13 universal primers (which anneal either side of the MCS of pTA131) using 5′ Taq Master Mix (Eppendorf) as per the manufacturer's directions. Clones were verified by Sanger sequencing using the UF sequencing facility. The specific primers used to generate each gene-deletion construct are detailed in Supplemental Table 3.

Once obtained, the confirmed deletion plasmids were passaged through a *dam^−^* strain of *Escherichia coli* (*Inv110*; Invitrogen, CA), transformed into *H. volcanii* H26 (or derivatives) as described above. Deletion of the targeted locus was selected for in a two-step process as described previously by Allers et al. [16]. Briefly, recombination of the deletion plasmid into the chromosome by a single cross-over event was selected for by growth on HvCA (i.e., in the absence of uracil). Subsequent excision of the integrated plasmid and target gene by a second recombination event was selected for by plating onto HvCA supplemented with uracil (10 *μ*g mL^−1^) and 5-fluoroorotic acid (5-FOA; 50 *μ*g mL^−1^). Gene deletion candidates were screened using a PCR-based method as follows. The forward primer was designed to anneal 5′ to the downstream fragment, and the reverse primer was designed to anneal within the upstream fragment. One pair of primers (denoted by the suffix _ext_Fwd and _ext_Rev) were designed to anneal within the flanking regions of the gene to be deleted and the amplicon size compared to wild-type and predicted sizes. To confirm loss of the gene, a second pair of primers (suffixed _int_Fwd and _int_Rev) was designed to anneal within the target gene to be deleted to confirm loss of the gene from the chromosome.

### 2.3. Other Plasmid Constructions

Target genes were amplified by PCR from purified *H. volcanii* genomic DNA using Phusion Hot Start polymerase (Finnzymes, Espoo, Finland), using primers listed in Supplementary Table 3. The amplicon was inserted between the *Nde*I and *Blp*I sites of pJAM202c [33].

### 2.4. *H. volcanii* tRNA Extraction and Analysis

Bulk tRNA was prepared, hydrolyzed, and analyzed by liquid chromatography-tandem mass spectrometry (LC-MS/MS) as described previously [34]. All tRNA purifications and analyses were performed independently at least twice.

### 2.5. High-Throughput Phenotype Analysis

For analysis of growth in liquid or on solid media, strains were routinely streaked from cryostorage onto Hv-YPC solid medium and incubated for 48 hrs at 44°C. Three tubes containing 5 mL of Hv-YPC were inoculated independently with single colonies and grown at 44°C for 48 hrs.

For monitoring of *H. volcanii* growth curves, 2 *μ*l of culture normalized to an OD_600_ of 1 were used to inoculate either 300 *μ*l of Hv-YPC or 300 *μ*l CDM. 250 *μ*l of inoculated medium was transferred to a 100-well plate. Growth curves were monitored in a Bioscreen-C Automated Growth Curve Analysis System (Growth Curves USA, MA) at 44°C with continuous shaking. Readings were recorded every 45 min (for clarity only one in 5 readings is shown in Figures 3(d) and 3(e)). For the duration of the experiments conducted here, no special measures were necessary to prevent evaporation/salt precipitation from the Bioscreen plate.

For analysis of growth on solid media, wild-type and mutant strains were grown at a range of temperatures and salt concentrations. 100 *μ*l of each culture grown in Hv-YPC was normalized to an OD_600_ of 1. This culture was serially diluted, and 10 *μ*l of the 10^−4^ dilution was plated onto a 96-well plate. Each well contained 100 *μ*l of Hv-YPC agar containing various salt concentrations. In addition to the standard final concentration of 18% salt water, as indicated in [12], the 30% salt water stock used to make standard Hv-YPC was diluted to achieve final concentrations of 12%, 14%, 23% and 25%. Each medium was duplicated so that the 6 salt concentrations occupied 12 wells (equating to a row on a 96-well plate). Each plate was replicated 5 times, and each incubated at 26°C, 30°C, 37°C, 44°C and 50°C, thus yielding a matrix of temperature and salt concentration. Depending on the temperature, plates were incubated 2 to 7 days. The top row of each plate was used for a wild-type control, and the various mutants were added to subsequent rows.

## 3. Results and Discussion

### 3.1. Accelerating Gene Deletion Constructions in *H. volcanii*

The deletion of a large number of genes from the *H. volcanii* genome required the development of a fast method to obtain deletion constructs. Construction of *H. volcanii* gene deletions can be divided into two phases: (1) generation of the deletion construct which involves established *E. coli*-based molecular biology, and (2) the introduction and selection of the correct gene-deletion candidate in the native organism. Phase 1 is hampered by the need to amplify by polymerase chain reaction (PCR) and clone multiple fragments of high GC content DNA (average 65% [14]). Phase 2 is time consuming inherent to the technique and organism; two independent recombination events must be selected for in a relatively slow-growing organism. Currently, described methods require cloning the up- and downstream regions flanking the gene to be deleted either together (making the three-way ligation reaction between the two fragments and linearized vector relatively inefficient), sequential cloning (which is time consuming), or to use proprietary topoisomerase-based techniques which can be expensive when applied to large gene sets [16, 17]. Thus, all existing techniques can require multiple rounds of cloning and as such are labor intensive and expensive, neither of which are desirable for the creation of large numbers of gene deletions. To expedite the generation of mutants, we explored the possibility of recombineering deletion constructs rather than employing traditional established molecular biology techniques using In-Fusion (Clontech, CA; [35]). Detailed methodology of this technique are included in the Materials and Methods and shown schematically in Figure 1. Utilization of this system enabled deletion constructs to be synthesized within 24 hrs of receiving the necessary oligonucleotides (compared to 2–4 weeks using previously described methods) and verified gene deletions in *H. volcanii* were achieved in 3–4 weeks. Furthermore, due to the apparent high accuracy of the technique, fewer deletion-construct candidates needed to be screened prior to introduction to *H. volcanii* reducing time spent and cost (generally three candidate plasmids were screened using a PCR-based method, of which all three were almost always correct). Although around a month is still far beyond the time required for model organisms of other kingdoms, this period is unavoidable due to the relative slow growth of the organism and for the two recombination events implicit in the deletion protocol. However, adoption of this method enables the construction of large numbers of gene deletion constructs in a realistic timeframe. Furthermore, the same technique could in principle be applied to rapidly generate plasmid constructs to manipulate the genome by insertions, such as for in-frame chromosomal protein-fusion tags although this remains untested at present.

Traditionally, the *H. volcanii* community has preferred deletion verification by Southern hybridization methods. Here, with the large number of putative mutants to screen, we have adopted the 2-fold PCR-based mutant verification process employed in *H. volcanii* by El Yacoubi et al. [17]. In addition to being at least as sensitive as Southern hybridizations, PCR is easily scalable. Two primers are designed to anneal outside of the affected gene, and two within. The two PCR reactions performed with these primers confirm alteration of the genomic region and are validated by comparison with the predicted amplicon size, and the primers landing within the gene absolutely confirm its absence when compared to the wild-type parent strain positive control. Generally, 36 candidates were screened which, in the cases mutants were achieved, typically yielded several deletions. The theoretical 50% mutant generation from resolution of the merodiploid was rarely achieved, possibly due to recombination restraints resulting from localized secondary structure. If gene deletions could not be identified, a further 100 candidates were screened, and if no mutants still were exhibited, the entire process was repeated at least once. 

**Figure 2:**
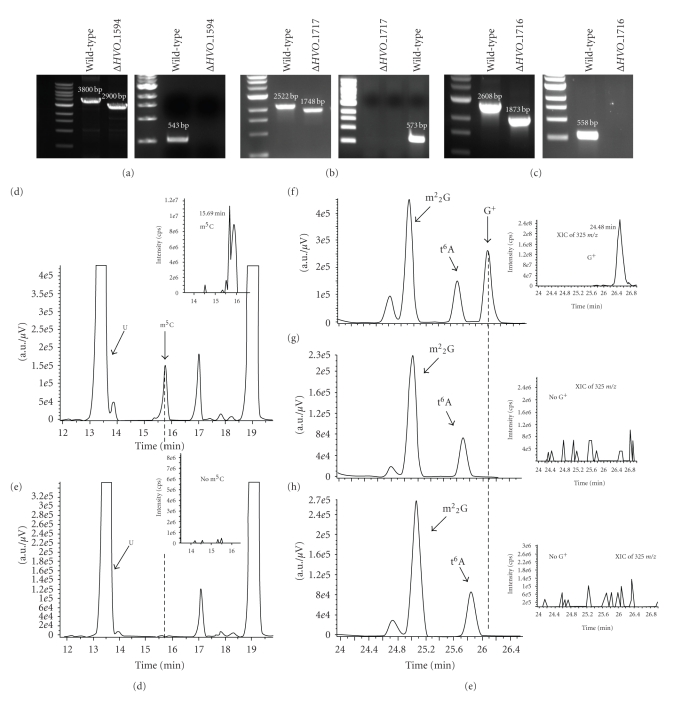
tRNA analysis. Gene deletions of (a) *HVO_1594*, (b) *HVO_1717* and (c) *HVO_1716* were verified by locus-specific PCR using primer pairs as indicated in Supplementary Table 3 designed to anneal outside and within the gene. Predicted amplicon sizes are indicated above each band. Analysis of LC-MS/MS tRNA extracted from *H. volcanii* strains showing the presence or absence of the m^5^C peak on the UV trace at 254 nm for H26 (d) and the corresponding extraction ion chromatogram for the H26 and Δ*HVO_1594* (e). Fragmentation analyses of m^5^C are shown as insets. LC-MS/MS analyses of tRNA extracted from (f) H26, (g) Δ*HVO_1717*, and (h) Δ*HVO_1716* indicate the presence and absence of G^+^. The peaks corresponding to m^2^_2_G and t^6^A are shown for comparison. Fragmentation analyses of G^+^ are shown as insets.

Using this protocol, 21 genes were successfully deleted out of 30 attempted (Table 1), and one strain was constructed containing two gene deletions (Δ*HVO_1105* Δ*HVO_2008*). To demonstrate the validity of our protocol, we further analyzed phenotypes of specific mutants. HVO_1594 was previously predicted to be responsible for the formation of the tRNA modification m^5^C on the basis of homology with *P. abyssi* protein PAB1947 [36], a modification shown to be present in almost all *H. volcanii* tRNA species [37]. *HVO_1594* was deleted from the chromosome using the method described above, and bulk tRNA from wild type and Δ*HVO_1594* cells grown in Hv-YPC media were analyzed by LC-MS/MS (Figure 2). As shown in Figure 2, the peak corresponding to m^5^C was absent from the tRNA extracted from the mutant. The identity of the corresponding peak from wild-type tRNA was confirmed by tandem mass fragmentation. Thus the LC-MS/MS analysis was consistent with the involvement of HVO_1594 in the formation of m^5^C in tRNA.

*HVO_1717* and *HVO_1716* are the respective homologs of bacterial *queE* and *queC* involved in the synthesis of preQ_0_, a precursor of the Queuosine modification found in bacterial tRNA [38, 39]. preQ_0_ is also a precursor in the biosynthesis of Archaeosine (G^+^) [40], a tRNA modification of the D-loop found in almost all archaeal tRNA species sequenced to date [41]. As these two modifications share the same precursor, a likely hypothesis is that HVO_1717 and HVO_1716 are involved in G^+^ biosynthesis. To test this, the corresponding gene deletion mutants were constructed as described in the Materials and Methods and, to avoid potential contamination of preQ_0_ from rich media [40], grown in defined media (CDM). The bulk tRNA extracted from these mutant strains, was purified and digested to ribonucleosides (Figure 2). Analysis by LC-MS/MS demonstrated that the peak corresponding to the G^+^ was absent in both of the mutant strains but present in the wild-type. Identity of the peak from the wild-type sample was confirmed by tandem mass fragmentation (Figure 2). These results confirmed that *HVO_1717* (*queE*) and *HVO_1716* (*queC*) are involved in G^+^ biosynthesis.

### 3.2. Discovering Essential Genes in *H. volcanii*

**Figure 3:**
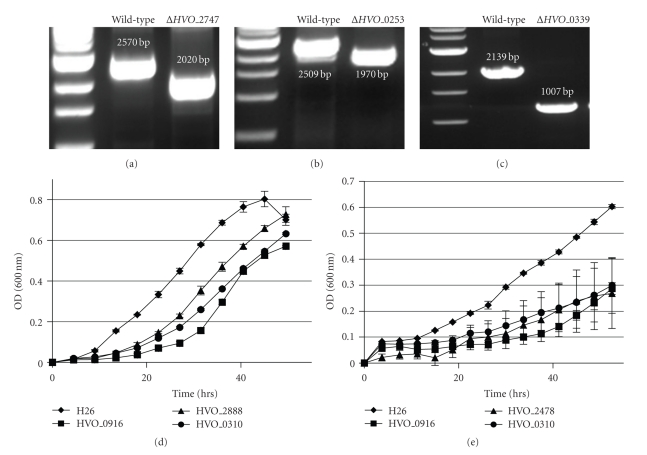
*H. volcanii* gene essentiality and growth analysis. Gene essentiality was confirmed by locus-specific PCR (a) *HVO_2747*, (b) *HVO_0253*, and (c) *HVO_0339*. Primers designed to anneal outside of the gene were used as detailed in Supplementary Table 3 and the amplicon size compared to the wild-type parent strain. Amplicon sizes are indicated beside each band. Growth analysis of *H. volcanii* H26 (wild-type) and the three most severe growth-defected strains in Hv-YPC media (d) and CDM (e). H26, HVO_0916, and HVO_0390 are indicated by diamonds, squares, and circles, respectively. Triangles indicate HVO_2888 (d) or HVO_2478 (e). Each point represents the mean of three independent biological repeats; error bars are plus and minus one standard error.

Using our techniques, we set out to delete 30 genes (Table 1). 22 strains were successfully constructed (Table 1); however, despite multiple attempts, eight genes could not be deleted (Table 1) raising the possibility that they were essential. To determine whether these were *bona fide* essential genes, and thus form the basis of the *H. volcanii* essential gene set alongside the likes of *cct1*, *hdaI*, and *pitA* [42–44], each of the eight genes were cloned into an *E. coli*/*H. volcanii* shuttle vector pJAM202. The clones were transformed into *H. volcanii* H26 derivatives whose chromosomes carried a verified integration of the corresponding gene deletion construct (see Section 2). With the specific genes expressed to high levels *in trans*, attempts were made again to delete the corresponding chromosomal gene copy. Deletion of the gene in this situation was checked as described in Materials and Methods, except that internal primers were not used as they would amplify a product regardless of successful deletion. Using this methodology, we successfully achieved deletion of the chromosomal copies of *HVO_0339*, *HVO_0253*, and *HVO_2747* suggesting that these genes are genuinely essential (Figures 3(a)–3(c)). Testing of the remaining five essential gene candidates is ongoing.

The essentiality of two of these three genes is expected. HVO_0339, a member of the COG1517 family, was recently shown *in vitro* to be responsible for the modification of cytidine to agmatidine at the wobble position of tRNA^Ile-^CAU [45, 46]. Agmatidine allows the modified tRNA^Ile-^CAU to decode AUA isoleucine codons and not methionine AUG codons [45, 46]. The functionally analogous modification in bacteria, lysidine, is essential [47]. HVO_0253 is a member of the YrdC/Sua5 family involved in the formation of N^6^-threonylcarbamoyl adenosine (t^6^A), a universal modification found at position 37 of tRNAs decoding ANN codons [48]. The corresponding gene family is essential in bacteria, and deletion of the yeast homologue (*YGL169W*) is viable but leads to severe growth phenotypes [48]. The essentiality of HVO_0253 reveals that concerning the role of t^6^A biosynthesis enzymes, Archaea are closer to bacteria than to eukaryotes. Finally, the function of HVO_2747, a member of COG1491, remains unknown, but its conservation in most Archaea, combined with the essentiality phenotype, makes it a top target for study. Our genetic results strongly suggest the three genes described above are essential but future investigation utilizing the previously described P_*TNA*_ promoter [44] will confirm this essentiality phenotype as well as determine the level of expression required for viability.

### 3.3. High-Throughput Growth Rate Analysis of *H. volcanii* Mutants

**Figure 4:**
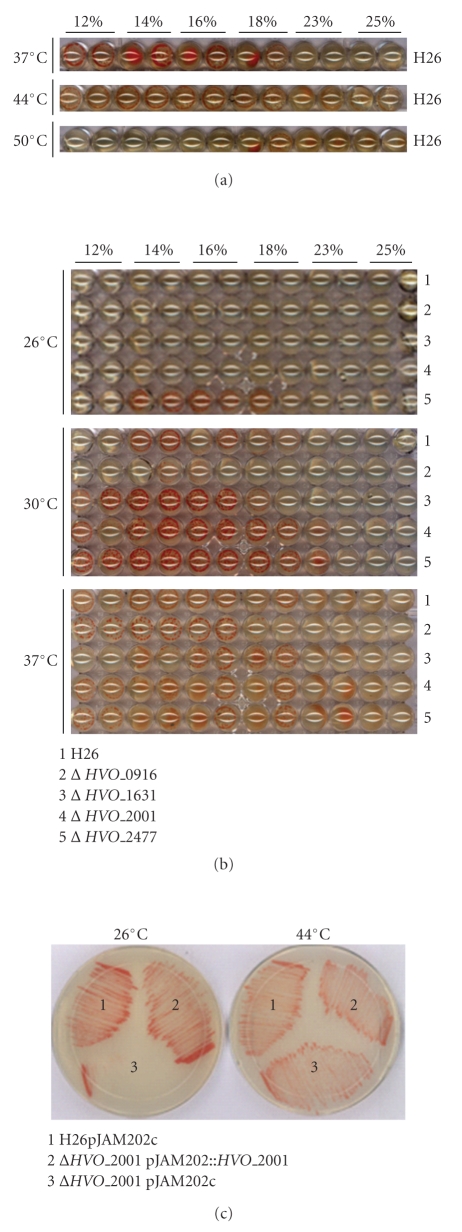
Salt and temperature tolerance of *H. volcanii* strains. ((a) and (b)) H26 (wild-type) and mutant strains (b) were subjected to high and low temperature and salt concentration. The salt concentration and temperature are indicated above each well. (c) H26 (1), Δ*HVO_2001* (2), and complemented Δ*HVO_2001* (3) streaked onto Hv-YPC and incubated at 26°C or 44°C as indicated.

Having deleted a relatively large number of genes, a second major goal of this study was to develop and implement high-throughput phenotyping and physiology approaches to an *H. volcanii*. Whilst these approaches have been applied to the analysis of *H. salinarum* [49], similar methods have not been performed with *H. volcanii*. Array-based phenotyping approaches (used to derive the “phenome”) have been employed in other model organisms to great success ( [50–52] and recently reviewed in [53]). Should a *H. volcanii* gene deletion library be constructed in the future, similar methods will be necessary for the functional assessment of a large number of mutants. We, therefore, initiated an investigation in which growth studies of wild-type and all mutant strains constructed in our laboratory in the last few years were compared in a range of conditions and media types. Our methods were designed to generate a large, quantitative dataset whilst maintaining a manageable, and easily scalable, labour load. We explored two approaches to strain phenotyping. The first approach was to perform growth curves in a 100-well format, and the second was determination of fitness of strains grown on solid media in a 96-well format. For growth curves, wild-type and 21 mutant strains were grown in rich (Hv-YPC) and defined media (CDM). Secondly, for solid media, those same strains were grown at a range of temperatures and salt concentrations. Each experiment contained at least two independent biological replicates. Using these techniques, we identified multiple novel phenotypes with one researcher in 2 weeks (summarized in Table 1, Figures 3 and 4 and Supplementary Table 4). 

A Bioscreen C apparatus was employed to obtain growth parameters of all strains in rich medium (Hv-YPC) and defined medium (CDM) to determine whether any gene deletions under investigation here were detrimental to growth. Many of the strains maintained growth rates that were indistinguishable from the wild type, however 7 mutants exhibited severe growth defects (Table 1 and Figures 3(d) and 3(e)). As would be expected, more mutants exhibited phenotypes when grown in defined (CDM) medium (Table 1). Δ*HVO_0390*, Δ*HVO_0916*, and Δ*HVO_2888* displayed the most severe growth defects in rich medium (Figures 3(d) and 3(e)). Δ*HVO_0916*, Δ*HVO_0390*, and Δ*HVO_2478* displayed the three most severe growth defects in defined medium (Figure 3). Complete growth-rate calculations of all strains in both media types are summarized in Table 1. Although the growth rate constants corresponding to doubling times of ~20 hrs are lower than has previously been reported [12, 13], this appears an artefact of using the Bioscreen C apparatus, where culture agitation, and therefore aeration, are significantly lower than in large cultures in a regular shaking incubator. As a control for this, H26 was grown in the same Hv-YPC media in regular culture flasks and determined to have a doubling time of ~4.5 hrs, strongly suggesting the differences are solely a result of the 100-well format used. HVO_0390 is a member of COG2016. The yeast homolog, Tma20, was identified as a ribosome-associated protein [54]. Loss of Tma20 caused a significant increase in ochre and opal codon read through in yeast [54]. The corresponding gene physically clusters with ribosomal protein genes in many archaeal genomes ([55] and see Tma20 subsystem on the SEED database http://theseed.uchicago.edu/FIG/SubsysEditor.cgi?page=ShowSpreadsheet&subsystem=DOE_COG2016). Therefore, although it remains untested at present, a role for HVO_0390 in maintaining translation fidelity in Archaea appears probable, and given this, the reduced growth rate of the corresponding deletion mutant is not surprising. HVO_0916 is homologous to Diphtine Synthase (Dph5), a methyltransferase of the diphtamide biosynthetic pathway. Diphtamide is a post translational modification found in Elongation factor 2 (EF2) in Archaea and eukaryotes and is the target of the diphtheria toxin [56–58]. Although the precise function of this modification remains unknown, deletion of genes in this pathway results in growth phenotypes and translation defects in yeast [59] and severely impaired development in mammals [60, 61]. Our analysis suggests that growth defects due to absence of diphtamide are also observed in Archaea, but this remains to be validated by confirming the absence of the modification in EF2 due to deletion of *HVO_0916*. These results suggest that obtaining growth curve analyses in a high-throughput fashion is a viable approach to study *H. volcanii* gene function and physiology.

### 3.4. High Throughput Phenotyping of *H. volcanii* Mutants

A key question in the analysis of halophiles, and Archaea at large, is to understand their ability to thrive in seemingly inhospitable environments. To begin to address this, and with the long-term intention of identifying the underlying genetic and molecular basis for this ability, the parent strain and mutants were subjected to growth at a range of temperatures (26°C–50°C) and salt concentrations (12%–25%). An interesting observation that was revealed by comparing large numbers of plates simultaneously is that *H. volcanii* salt tolerance appears to be dependent on temperature. At relatively low temperatures (26°C–37°C), there is a clear preference for relatively high salt concentrations whereas at higher temperature (50°C), salt tolerance decreases, and high salt concentration becomes inhibitive to growth (Figure 4(a)). Comparison of growth of wild-type to mutant strains across the various salt concentrations and temperatures revealed several strains that exhibit cold sensitivities. These included deletions in HVO_0916, HVO_1631, and HVO_2001 (Figure 4(b) and Supplementary Table 4). Conversely, the strain bearing a deletion in HVO_2477 exhibits increased tolerance to low temperature. 

As often seen when deleting components of the translation apparatus [62, 63], deleting *HVO_0916, HVO_1631*, and *HVO_2001* gave rise to cold sensitivity phenotypes. As discussed above, HVO_0916 is homologous to the diphthamide biosynthesis protein Dph5 and HVO_1631 is homologous to Dph2, the first enzyme of the pathway [64]. *HVO_2001* encodes a tRNA-guanine–transglycosylase (TGT) [17]. TGT is a key enzyme in the synthesis of Archaeosine (G^+^), a modification of tRNA found specifically in Archaea. G^+^ is found at the elbow of the D-arm and T-arm at position 15 of almost all archaeal tRNA bearing a guanine at this position. We had previously shown that Δ*HVO_2001* was viable and lacked G^+^ in tRNA [17]. The results here show a first growth phenotype for an Archaeon lacking Archaeosine, cold sensitivity. In eukaryotic and bacterial tRNA, position 15 is part of the Mg^2+^ binding site [65, 66]. Mg^2+^ ions induce folding and maintain the tRNA tertiary structure (16, 43, 48, and 52). Also, Oliva et al. showed that *in silico*, Mg^2+^ and G^+^ play an interchangeable role in stabilizing the G15-C48 Reverse Watson-Crick geometry [67]. Together, these studies imply that G^+^ might play a role in inducing folding and maintaining the structural integrity of tRNA. Therefore, the absence of the G^+^ modification could confer more rigidity to tRNA and perturb folding at low temperatures explaining the cold-sensitivity phenotype. To independently confirm the 96-well plate assays, we reproduced the Δ*HVO_2001* phenotype on 100 mm petri dishes (Figure 4(c)) and complemented the cold-sensitivity phenotype by expressing the *HVO_2001 in trans*. As HVO_2001, HVO_1717, and HVO_1716 form part of the same pathway for G^+^ biosynthesis, the same cold-sensitivity phenotype might also be expected in strains bearing deletions of *HVO_1717* and *HVO_1716*. However, we did not observe a cold-sensitivity phenotype for these two genes. An explanation for this observation is the possibility of preQ_0_ salvage from rich media (on which these phenotype screens were performed). Therefore, in these strains G^+^ is still made. This hypothesis could be tested by repeating these assays on defined medium, where salvage of the PreQ_0_ base would not occur.

In conclusion, we have employed modern recombineering-based methods to rapidly generate gene-deletion constructs, ultimately resulting in the construction of 22 strains. Eight genes included in our pipeline could not be deleted by these methods; we genetically demonstrated provided evidence for the probable essentiality of 3 of those. Furthermore, using array-based phenotyping methods, we were able to identify multiple growth phenotypes of the mutants analyzed using methods that could easily be scaled to encompass much larger gene-deletion collections. The work presented here paves a potential road for the systematic creation and analysis of a global *H. volcanii* gene-deletion library, which would support the genetic analysis of the plethora of hypothetical and poorly annotated genes in Archaea.

## Authors' Contributions

IKB and VdC-L designed & performed research and wrote the manuscript. GP, CEB, KG and BEY helped with strain construction. GP, CEB and BEY helped with the design of the phenotypic analysis.

## Supplementary Material

Strains, plasmids and oligonucleotides used in this study are detailed in Supplementary Material Tables 1, 2 and 3 respectively. Salt and temperature tolerance phenotypes of wild-type and each mutant strain are shown in Supplementary Material Table 4.

## Figures and Tables

**Table 1:** Gene-deletion summary.

Gene	COG	Strain growth rate Hv-YPC^a^	Strain growth rate CDM^a,b^	Phenotype/essentiality verified
H26 (wild-type)	—	0.0227	0.0133	—
*HVO_0156*	1041	0.0223	0.0105	
*HVO_0658*	0585	0.0281	0.0175	
*HVO_2001*	0343	0.0233	0.0123	Cold-sensitivity
*HVO_0236*	1867	0.0238	0.0104	
*HVO_2906*	0565	ND^c^	ND^c^	
*HVO_2888*	1243	0.0102	0.0115	Severe growth defect
*HVO_0580*	0037	0.0271	0.0124	
*HVO_2736*	1444	0.0210	0.0115	(20)
*HVO_1852*	0101	0.0151	0.0151	Growth defect
*HVO_1594*	0144	0.0107	0.0107	Growth defect
*HVO_0916*	1798	0.0055	0.0088	Cold-sensitivity, severe growth defect
*HVO_1631*	4112	0.0196	0.0097	Cold-sensitivity, slight growth defect
*HVO_2477*	2890	0.0219	0.0117	Cold-resistance
*HVO_1716*	0603	0.022	0.0143	
*HVO_1717*	0602	0.020	0.0118	
*HVO_B0354*	1357	0.0266	0.0128	
*HVO_0390*	2016	0.0066	0.0064	Severe growth defect
*HVO_2478*	0042	0.0243	0.0075	Defined media growth defect
*HVO_1475*	2263	0.0266	0.0177	
*HVO_1105*	1374	0.0216	0.0124	
*HVO_2008*	1549	0.0245	0.0154	
*HVO_1105*	1374,	0.025	0.0127	
*HVO_0574*	2226	Not deleted		Predicted essential
*HVO_0339*	1571	Not deleted		Deleted only when gene present *in trans *
*HVO_0697*	2047	Not deleted		Predicted essential
*HVO_0253*	0009	Not deleted		Deleted only when gene present *in trans *
*HVO_1173*	1303	Not deleted		Predicted essential
*HVO_1383*	2519	Not deleted		Predicted essential
*HVO_2747*	1491	Not deleted		Deleted only when gene present *in trans *
*HVO_0929*		Not deleted		Predicted essential

^a^growth rates are presented hr^−1^.

^b^0.4% (v/v) glycerol used as a carbon source.

^c^Not determined.
