# Erratum to “Towards a Systems Approach in the Genetic Analysis of Archaea: Accelerating Mutant Construction and Phenotypic Analysis in *Haloferax volcanii*”

**DOI:** 10.1155/2011/867406

**Published:** 2011-12-28

**Authors:** Ian K. Blaby, Gabriela Phillips, Crysten E. Blaby-Haas, Kevin S. Gulig, Basma El Yacoubi, Valérie de Crécy-Lagard

**Affiliations:** Department of Microbiology and Cell Science, University of Florida, Gainesville, FL 32611-0700, USA

In Page 6, right column, second paragraph, COG1517 should be COG1571.

In Table  1, page 7, *HVO_2477* should be *HVO_2744* and *HVO_B0354* should be *HVO_B0357*.

In Page 8, right column, end of last paragraph, HVO_2477 should be HVO_2744.

In Supplemental Table  1, the entry VDC2399 H26 Δ*HVO_B0354 *should read VDC2399 H26 Δ*HVO_B0357.*

In Supplemental Table  4, all *HVO_2477* entries should be *HVO_2744*, and all *HVO_B0354* entries should be *HVO_B0357*.

These corrections do not influence the overall conclusions of this study.

