# Supplementary material for: Towards a Systems Approach in the Genetic Analysis of Archaea: Accelerating Mutant Construction and Phenotypic Analysis in Haloferax volcanii
Source: Archaea. 2010 Dec 23;2010:426239. doi: 10.1155/2010/426239 (PMC3017900; doi:10.1155/2010/426239)
Supplement: Supplementary file 1 — Strains, plasmids and oligonucleotides used in this study are detailed in Supplementary Material Tables 1, 2 and 3 respectively. Salt and temperature tolerance phenotypes of wild-type and each mutant strain are shown in Supplementary Material Table 4. [file 426239.f1.pdf]

Supplemental Table 1 Strains

**Strain**	**Genotype**	**Reference**
E. coli Top10 DH5a	F⁻ *mcr*A Δ(*mrr-hsd*RMS-*mcr*BC) φ80*lac*ZΔM15 Δ*lac*X74 *rec*A1 *ara*D139 Δ(*ara-leu*) 7697 *gal*U *gal*K *rps*L (Str^R) *end*A1 *nup*G λ-	Invitrogen, CA
E. coli inv110	F′ {*traΔ36 proAB lac*I_q *lacZΔM15*} *rps*L (Str^R) *thr leu end*A *thi*-1 *lacY galK galT ara ton*A *tsx dam dcm supE44 Δ(lac-proAB)* Δ*(mcr*C-*mrr)102*::Tn*10* (Tet^R)	Invitrogen, CA
H. volcanii DS70		[5]
H. volcanii H26	DS70 Δ***pyrE2***	[1]
VDC2177	H26 Δ*HVO_2736*	[2]
VDC3226	H26 pJAM202c	This work
VDC2329	H26 Δ*HVO_1105*	This work
VDC2347	H26 Δ*HVO_0156*	This work
VDC2364	H26 Δ*HVO_2493*	This work
VDC2385	H26 Δ*HVO_0236*	This work
VDC2399	H26 Δ*HVO_B0354*	This work

VDC2414	H26 Δ*HVO_0390*	This work
VDC2452	H26 Δ*HVO_2888*	This work
VDC2457	H26 Δ*HVO_0580*	This work
VDC2459	H26 Δ*HVO_1852*	This work
VDC2469	H26 Δ*HVO_1475*	This work
VDC2476	H26 Δ*HVO_2478*	This work
VDC2506	H26 Δ*HVO_0658*	This work
VDC2514	H26 Δ*HVO_2744*	This work
VDC2517	H26 Δ*HVO_0916*	This work
VDC2527	H26 Δ*HVO_1631*	This work
VDC2545	H26 Δ*HVO_2906*	This work
VDC3241	H26 Δ*HVO_2001*	[2]
VDC3347	H26 Δ*HVO_1717*	This work
VDC3352	H26 Δ*HVO_1716*	This work
VDC3259	H26 Δ*HVO_2001* pJAM202c	This work
VDC3265	H26 Δ*HVO_2001* pGP109	This work
VDC3414	H26 Δ*HVO_1105* Δ*HVO_2008*	This work
VDC5203	H26 Δ*HVO_2008*	This work
VDC2554	H26 Δ*HVO_0253* pIKB437	This work
VDC2552	H26 Δ*HVO_2747* pIKB434	This work
VDC2556	H26 Δ*HVO_0339* pIKB434	This work

Supplemental Table 2 Plasmids

Plasmid	Description	Reference
pJAM202	*H. volcanii* shuttle vector	[3]
pTA131	Amp^R; ColE1	[1]
pBY158	pTA131 derivative containing the $attCm^RCcd^R$ cassette	[2]
pBY165.31	pTA131 Δ*HVO_2001*	[2]
pNAB135	pTA131 Δ*HVO_2008*	[4]
pGP068	pBY158 Δ*HVO_2747*	This work
pGP041	pCR8/GW 1000BpUpDn Δ *HVO_0929*	This work
pGP019	pCR8/GW 1000BpUPDn *HVO_0929*	This work
pIKB264	pTA131 Δ*HVO_1383*	This work
pIKB103	pTA131 Δ*HVO_2736*	[2]
pIKB154	pTA131 Δ*HVO_0156*	This work
pIKB156	pTA131 Δ*HVO_0574*	This work
pIKB162	pTA131 Δ*HVO_1105*	This work
pIKB172	pTA131 Δ*HVO_1594*	This work
pIKB180	pTA131 Δ*HVO_1979*	This work
pIKB184	pTA131 Δ*HVO_1173*	This work
pIKB199	pTA131 Δ*HVO_1852*	This work

pIKB203	pTA131 Δ*HVO_2888*	This work
pIKB207	pTA131 Δ*HVO_1475*	This work
pIKB211	pTA131 Δ*HVO_0697*	This work
pIKB215	pTA131 Δ*HVO_0236*	This work
pIKB219	pTA131 Δ*HVO_2493*	This work
pIKB229	pTA131 Δ*HVO_0339*	This work
pIKB240	pTA131 Δ*HVO_0580*	This work
pIKB251	pTA131 Δ*HVO_2906*	This work
pIKB262	pTA131 Δ*HVO_B0354*	This work
pIKB265	pTA131 Δ*HVO_0253*	This work
pIKB268	pTA131 Δ*HVO_1717*	This work
pIKB271	pTA131 Δ*HVO_3412*	This work
pIKB274	pTA131 Δ*HVO_0390*	This work
pIKB276	pTA131 Δ*HVO_1716*	This work
pIKB290	pTA131 Δ*HVO_1173*	This work
pIKB304	pTA131 Δ*HVO_2478*	This work
pIKB315	pTA131 Δ*HVO_0658*	This work
pIKB415	pTA131 Δ*HVO_1631*	This work
pIKB417	pTA131 Δ*HVO_0916*	This work
pIKB418	pTA131 Δ*HVO_2744*	This work
pIKB371	*HVO_0339* inserted between the *Nde*I *Blp*I sites of pJAM202c	This work

pIKB437	*HVO_0253* inserted between the *Nde*I *Blp*I sites of pJAM202c	This work
pIKB367	*HVO_1979* inserted between the *Nde*I *Blp*I sites of pJAM202c	This work
pIKB434	*HVO_2747* inserted between the *Nde*I *Blp*I sites of pJAM202c	This work
pGP109	*HVO_2001* inserted between the *Nde*I and *Blp*I sites of pJAM202	This work

Supplemental Table 3 Oligonucleotides

Oligonucleotide	Sequence	Purpose
**Deletion constructs using In-fusion**		
HVO_1631_NF	cgggcccccctcgaggaggaagaccgtcgaaacc	Construction of *HVO_1631* deletion construct, pIKB415
HVO_1631_NR	gacgcgttcatatgcgttccacaagccgatgct	
HVO_1631_CF	gcatatgaacgcgtctcgacgatctgttcgagttc	
HVO_1631_CR	cgggctgcaggaattcgtaatcgcgccctgactg	
HVO_0916_NF	cgggcccccctcgagcatcgaatcactcggctaca	Construction of *HVO_0916*
HVO_0916_NR	gacgcgttcatatgcgtccaccacgtcgaagc	

HVO_0916_CF	gcatatgaacgcgtccctcccgcccttcgac	deletion construct, pIKB417
HVO_0916_CR	cgggctgcaggaattcaggatgccgccttcgag	
HVO_2744_NF	cgggcccccccctcgagacggcgtcgaggaactc	Construction of *HVO_2744* deletion construct, pIKB418
HVO_2744_NR	gacgcgttcatatgcgaggtcgtcgttcaggagtc	
HVO_2744_CF	gcatatgaacgcgtcggctggtagaccgaggtgtt	
HVO_2744_CR	cgggctgcaggaattctctcccgaccgttcgaaat	
HVO_2478_N_Fwd	cgggcccccccctcgaggaacgtgcgggcgaag	Construction of *HVO_2478* deletion construct, pIKB304
HVO_2478_N_Rev	gacgcgttcatatgccgacgaaagcgaggtgtc	
HVO_2478_C_Fwd	gcatatgaacgcgtcgctgaggctcgcaacg	
HVO_2478_C_Rev	cgggctgcaggaattcgtccacatcactcgcacct	
HVO_1716_N_Fwd	cgggcccccccctcgagcgacgacgactaccacctg	Construction of *HVO_1716* deletion construct, pIKB276
HVO_1716_N_Rev	gacgcgttcatatgcgacgtgggttcggtagtca	
HVO_1716_C_Fwd	gcatatgaacgcgtcgcgacccgattccgta	
HVO_1716_C_Rev	cgggctgcaggaattcgttgtcctcgaaggcgaag	
HVO_1717_N_Fwd	cgggcccccccctcgaggagcgacctagaacgacacc	Construction of *HVO_1717* deletion construct, pIKB268
HVO_1717_N_Rev	gacgcgttcatatgcgcgaacagctcgtttatcg	
HVO_1717_C_Fwd	gcatatgaacgcgtccgactccacgtcgacctct	
HVO_1717_C_Rev	cgggctgcaggaattcctcgaaggcgtcgaagaact	

HVO_339_N_IfKO_Fwd	cgggccccccctcgagtcgaggctcacgtacagtcc	Construction of *HVO_339* deletion construct, pIKB229
HVO_339_N_IfKO_Rev	gacgcgttcatatgccgccagataggtcgtacaca	
HVO_339_C_IfKO_Fwd	gcatatgaacgcgtcatcgctaagccgctcgtt	
HVO_339_C_IfKO_Rev	cgggctgcaggaattcacggtccatgctcgatatgt	
HVO_1475_N_IfKO_Fwd	cgggccccccctcgagagtcgacgacgaccaacac	Construction of *HVO_1475* deletion construct, pIKB207
HVO_1475_N_IfKO_Rev	gacgcgttcatatgcagttcgacttccacgacgag	
HVO_1475_C_IfKO_Fwd	gcatatgaacgcgtcggtactgttcgagcgagacct	
HVO_1475_C_IfKO_Rev	cgggctgcaggaattcgaacccaccccgttagatta	
HVO_0697_N_IfKO_Fwd	cgggccccccctcgagcccttggaggtcgactacg	Construction of *HVO_0697* deletion construct, pIKB211
HVO_0697_N_IfKO_Rev	gacgcgttcatatgctcaggagatgcaacagcaac	
HVO_0697_C_IfKO_Fwd	gcatatgaacgcgtccagcccctcgatgaggac	
HVO_0697_C_IfKO_Rev	cgggctgcaggaattcgtcccctacgtcaacgtcac	
HVO_0253_N_IfKO_Fwd	cgggccccccctcgaggtcaacgcggagttctgg	Construction of *HVO_0253* deletion construct, pIKB265
HVO_0253_N_IfKO_Rev	gacgcgttcatatgcgtctcggtcgggtagacg	
HVO_0253_C_IfKO_Fwd	gcatatgaacgcgtcgacatcgaggcgtggttaga	
HVO_0253_C_IfKO_Rev	cgggctgcaggaattcgtcgcgcacaaactcgtc	
HVO_0236_N_IfKO_Fwd	cgggccccccctcgaggtgagccgtgtgcttacaga	Construction of *HVO_0236* deletion
HVO_0236_N_IfKO_Rev	gacgcgttcatatgccctgtgtggggttgtagaaga	
HVO_0236_C_IfKO_Fwd	gcatatgaacgcgtcgctgagatacgcgaggtgac	

HVO_0236_C_IfKO_Rev	cgggctgcaggaattcgcgagagcagttggagaaac	construct, pIKB215
HVO_2493_N_IfKO_Fwd	cgggccccccctcgagcagagagcgacagttgacga	Construction
HVO_2493_N_IfKO_Rev	gacgcgttcatatgccgtctgtctgggcgaact	of *HVO_2493*
HVO_2493_C_IfKO_Fwd	gcatatgaacgcgtcgcttgtcgaggttgacgac	deletion
HVO_2493_C_IfKO_Rev	cgggctgcaggaattcgcacccagcatggatatgtt	construct, pIKB219
HVO_574_N_IfKO_Fwd	cgggccccccctcgagcgacggcgagcagttc	Construction
HVO_574_N_IfKO_Rev	gacgcgttcatatgcgatttcggtcatctcgggta	of *HVO_0574*
HVO_574_C_IfKO_Fwd	gcatatgaacgcgtccgaggatgcgctcgac	deletion
HVO_574_C_IfKO_Rev	cgggctgcaggaattcgcgcaggcgaacctc	construct, pIKB156
HVO_1979_N_IfKO_Fwd	cgggccccccctcgagccgacgatggcgtgtt	Construction
HVO_1979_N_IfKO_Rev	gacgcgttcatatgcaggacgagccgttcgag	of *HVO_1979*
HVO_1979_C_IfKO_Fwd	gcatatgaacgcgtcagacaggcgtcgcagacc	deletion
HVO_1979_C_IfKO_Rev	cgggctgcaggaattccggagaacagaagcgagaag	construct, pIKB180
HVO_1105_N_IfKO_Fwd	cgggccccccctcgagggccatgccgtagagacc	Construction
HVO_1105_N_IfKO_Rev	gacgcgttcatatgcccggaggtcgtccaaatc	of *HVO_1105*
HVO_1105_C_IfKO_Fwd	gcatatgaacgcgtccgcggacgaagagtgag	deletion
HVO_1105_C_IfKO_Rev	cgggctgcaggaattcgagaacgctgggcgaag	construct, pIKB162
HVO_1383_N_IfKO_Fwd	cgggccccccctcgagagccgacgccgacac	Construction

HVO_1383_N_IfKO_Rev	gacgcgttcatatgcccgtggacgaggagtatcac	of *HVO_1383* deletion construct, pIKB
HVO_1383_C_IfKO_Fwd	gcatatgaacgcgtccaccggctacctcgtgtt	
HVO_1383_C_IfKO_Rev	cgggctgcaggaattccgtggcgctttctgttc	
HVO_0658_N_IfKO_Fwd	cgggccccccctcgagcgctcgtggccatcttc	Construction of *HVO_0658* deletion construct, pIKB315
HVO_0658_N_IfKO_Rev	gacgcgttcatatgcgacgacctcgtcttcgactt	
HVO_0658_C_IfKO_Fwd	gcatatgaacgcgtccgcacgcggaagtcct	
HVO_0658_C_IfKO_Rev	cgggctgcaggaattccacgagcgcgacgac	
HVO_1173_N_IfKO_Fwd	cgggccccccctcgagagcttggaggagccgtagat	Construction of *HVO_1173* deletion construct, pIKB265
HVO_1173_N_IfKO_Rev	gacgcgttcatatgcgacgtgcgtcgtcatcc	
HVO_1173_C_IfKO_Fwd	gcatatgaacgcgtcccgaaaaccgggtggt	
HVO_1173_C_IfKO_Rev	cgggctgcaggaattcgaggtcgtcgttgtccatc	
HVO_1852_N_IfKO_Fwd	cgggccccccctcgaggcgcgctcgatgtagtg	Construction of *HVO_1852* deletion construct, pIKB199
HVO_1852_N_IfKO_Rev	gacgcgttcatatgcatccgcgagggagtcg	
HVO_1852_C_IfKO_Fwd	gcatatgaacgcgtccggctgtcgctggaac	
HVO_1852_C_IfKO_Rev	cgggctgcaggaattcgtttccgcccgtcgta	
HVO_2906_N_IfKO_Fwd	cgggccccccctcgagcgacaccgctactttctcca	Construction of *HVO_2906* deletion construct,
HVO_2906_N_IfKO_Rev	gacgcgttcatatgcgctgacgggcgtgttc	
HVO_2906_C_IfKO_Fwd	gcatatgaacgcgtcgccgtgttggcggtatc	
HVO_2906_C_IfKO_Rev	cgggctgcaggaattcccgagatattcaccgacgag	

		pIKB251
HVO_0156_N_IfKO_Fwd	cgggccccccctcgagagcgacatcgaactctcg	Construction of *HVO_0156* deletion construct, pIKB154
HVO_0156_N_IfKO_Rev	gacgcgttcatatgcgtgcggtacgtgcatctg	
HVO_0156_C_IfKO_Fwd	gcatatgaacgcgtccccgaccagttcgacgac	
HVO_0156_C_IfKO_Rev	cgggctgcaggaattccgagagtggaactgcgagtc	
HVO_1594_N_IfKO_Fwd	cgggccccccctcgaggatgcccgagaggaacac	Construction of *HVO_1594* deletion construct, pIKB172
HVO_1594_N_IfKO_Rev	gacgcgttcatatgcaaaaaggcctcctcgtcgt	
HVO_1594_C_IfKO_Fwd	gcatatgaacgcgtccgtctacccgcaccacaac	
HVO_1594_C_IfKO_Rev	cgggctgcaggaattctattccggtttgacgaggtc	
HVO_2747_N_IfKO_Fwd	cgggccccccctcgagccgaactccttttggaacat	Construction of *HVO_2747* deletion construct, pGP035
HVO_2747_N_IfKO_Rev	gacgcgttcatatgcgaaggaggtcatcgtcgag	
HVO_2747_C_IfKO_Fwd	gcatatgaacgcgtcgtggcggagtacgtcgag	
HVO_2747_C_IfKO_Rev	cgggctgcaggaattccgcttcaacggtctcacc	
ORF02092_N_IfKO_Fwd	cgggccccccctcgagcgacggcgagcagttc	Construction of *HVO_2888* deletion construct, pIKB207
ORF02092_N_IfKO_Rev	gacgcgttcatatgcgatttcggtcatctcgggta	
ORF02092_C_IfKO_Fwd	gcatatgaacgcgtccgaggatgcgctcgac	
ORF02092_C_IfKO_Rev	cgggctgcaggaattcgcgcaggcgaacctc	
HVO_580_N_IfKO_Fwd	cgggccccccctcgagcgccgtttcggagctt	Construction of *HVO_0580*
HVO_580_N_IfKO_Rev	gacgcgttcatatgcgcacttgtcgcactccatt	

HVO_580_C_IfKO_Fwd	gcatatgaacgcgtcggaagtgccgactcatcg	deletion construct, pIKB240
HVO_580_C_IfKO_Rev	cgggctgcaggaattcgtggcctcgtccacatcac	
HVO_B0357_N_Fwd	cgggcccccctcgagagcgcgaggtcgtactcc	Construction of *HVO_B0357* deletion construct, pIKB262
HVO_B0357_N_Rev	gacgcgttcatatgcccgcgaggaacactacgg	
HVO_B0357_C_Fwd	gcatatgaacgcgtccgtgatgacgtactgggtga	
HVO_B0357_C_Rev	cgggctgcaggaattccgacgactggtacgagaaca	
HVO_0339_N_Fwd	cgggcccccctcgagtctatctccaccggtccttg	Construction of *HVO_0390* deletion construct, pIKB274
HVO_0339_N _Rev	gacgcgttcatatgcgcgggacttgaccttcatac	
HVO_0339_C _For	gcatatgaacgcgtccgacgacctcttcgagttct	
HVO_0339_C _Rev	cgggctgcaggaattcgctgacgaacgtcggact	
Deletion constructs, synthesised using methods other than In-Fusion		
tgtA2HvUPF	tctagaggtgattaccacgcgcaag	Construction of *HVO_2008* deletion plasmid, described in [4]
tgtA2HvUPR	ggatccgcgtgaacctcgaagtagt	
tgtA2HvDSol1	aatggatcctcgaagggtaattcgg	
tgtA2HvDSol2	aatgcggccgcaagcttcggcagtcgcgtgttgatg	
UpDn_HVOB2736_fwd	ctg gag gat gtc gcc ctc gc	Construction

UpDn_HVO2736_rev	gac ggt cag gtg cgc ccc	of *HVO_2736* deletion plasmid, described in [2]
RevPCR_HVO2736_fwd	gacgcggcccgcgaggaa	
RevPCR_HVO2736_rev	ttcggtcatgggatggatac	
Gene deletion verification		
HVO_2478_Int_Fwd	gaggtactcggtcgctgtct	Internal checks for *HVO_2478*
HVO_2478_Int_Rev	ggattccctcgacgagttc	
HVO_2478_Ext_Fwd	acgtcgtacagcgtgcatc	External checks for *HVO_2478*
HVO_2478_Ext_Rev	gacgtggacaccgtcctct	
HVO_1105_Int_F	ccgacgcagacatcgaact	Internal checks for *HVO_1105*
HVO_1105_Int_R	ccgtctgaaagtcgagcatc	
HVO_1105_fwd	gcactgttgaacgacctcct	External checks for *HVO_1105*
HVO_1105_rev	ctgtcggagcgaaaagagac	
HVO_B0357_Int_F	aggtcgtacgtctcgttcgt	Internal checks for *HVO_B0357*
HVO_B0357_Int_R	ggtcgggctgtacttcttca	
HVO_B0357_Ext_F	cggacagtcgtatgtgcaac	External checks for
HVO_B0357_Ext_R	cgacgactggtacgagaaca	

		HVO_B0357
HVO_1594_int_Fwd	gcatcctggaactcgaagac	Internal checks for *HVO_1594*
HVO_1594_int_Rev	aggtgcaggtggagtagacg	
HVO_1594_ext_Fwd	cggcttaaacgtgtagactgc	External checks for *HVO_1594*
HVO_1594_ext_Rev	ggactcagttcgatgacgttc	
HVO_0253_Int_F	aacgcgtcttcgacatcaa	Internal checks for *HVO_0253*
HVO_0253_Int_R	gtcgtcgtgctcgtctaacc	
HVO_0253_Ext_F	cgggaccggaattaagagtt	External checks for *HVO_0253*
HVO_0253_Ext_R	tgccatgtcagtaagccaag	
HVO_2493_Int_Fwd	cgtctttgaggtcgtggag	Internal checks for *HVO_2493*
HVO_2493_Int_Rev	cgaaggctcgaaagagtacg	
HVO_2493_Ext_Fwd	gttcctcggctactccgtct	External checks for *HVO_2493*
HVO_2493_Ext_Rev	gcctctgacagcagcaaata	
HVO_1173_int_Fwd	gggtcatcctaccggacaac	Internal checks for *HVO_1173*
HVO_1173_int_Rev	gccgggacgactttcttg	
HVO_1173_Ext_fwd	gcgataccgacgaggagtc	External

HVO_1173_Ext_rev	ggtcctcgtagcagtccatc	checks for *HVO_1173*
HVO_1852_Int_F	cgaactcgacttccgggta	Internal checks for *HVO_1852*
HVO_1852_Int_R	gtgtcccgactggtgtacg	
HVO_1852_Ext_fwd	gagtacctcggcctcctctc	External checks for *HVO_1852*
HVO_1852_Ext _rev	ctccaactcaggagcgaaag	
HVO_2888_Int_F	cggaagtcctccagacacat	Internal checks for *HVO_2888*
HVO_2888_Int_R	tcgaacagcttcgacacatc	
HVO_2888_Ext_fwd	gaagtcgaagtgggcgacta	External checks for *HVO_2888*
HVO_2888_Ext _rev	ctcggctacgagacgaagag	
HVO_0580_Int_Fwd	ctcttaccgcgagtttcacc	Internal checks for *HVO_0580*
HVO_0580_Int_Rev	accgcgaacttctccagtt	
HVO_0580_Fwd	ttcgcgatatgctcaatgaa	External checks for *HVO_0580*
HVO_0580_Rev	cgcggtagagttcggagtag	
HVO_0339_Int_F	ctcttaccgcgagtttcacc	Internal checks for *HVO_0339*
HVO_0339_Int_R	accgcgaacttctccagtt	

HVO_0339_Ext_F	gttcatgccgttttcgtact	External checks for *HVO_0339*
HVO_0339_Ext_R	cgacagtcatcgtgaaatcg	
HVO_0697_Int_F	gttgctgttgcatctcctga	Internal checks for *HVO_0697*
HVO_0697_Int_R	ctaccacctcaccgacacct	
HVO_0697_Ext_F	cccgacgtagtgttccatct	External checks for *HVO_0697*
HVO_0697_Ext_R	gacaagccgacgaagaagac	
HvO_1383_Int_F	cggacctcttcaaccacttc	Internal checks for *HVO_1383*
HvO_1383_Int_R	tcgttgaagtccatctgtcg	
HvO_1383_Ext_fwd	gctagcacggctcctacaac	External checks for *HVO_1383*
HvO_1383_Ext_rev	cggtcgtgaactcctctttc	
HVO_0156_Int_Fwd	ggtacttgacgaagccgaag	Internal checks for *HVO_0156*
HVO_0156_Int_Rev	acgctgtccaaggagttcag	
HVO_0156_Ext_Fwd	gatgcagcagatgcacgta	External checks for *HVO_0156*
HVO_0156_Ext_Rev	gacgtgcgaggactcacc	
HVO_0658_Int_fwd	gtggagaccgactgtgtgg	Internal checks for
HVO_0658_Int_rev	cgaactcgactcgctgaac	

		HVO_0658
HVO_0658_Ext_fwd	gttcgggtggatacaggaga	External checks for *HVO_0658*
HVO_0658_Ext_rev	agaactcgtggtggagttgc	
HVO_0236_Int_Fwd	caacgccctcatgtacgac	Internal checks for *HVO_0236*
HVO_0236_Int_Rev	ccgaagtcctctgtcacctc	
HVO_0236_Ext_fwd	cggaccggaacatctcag	External checks for *HVO_0236*
HVO_0236_Ext_rev	gtacgagcatacccgttcgt	
HVO_2906_Int_FWD	gagcaactcgtcgaaaaagc	Internal checks for *HVO_2906*
HVO_2906_Int_Rev	acttcggcatctacgacctc	
HVO_2906_Ext_fwd	agttcttgcggttgatggag	External checks for *HVO_2906*
HVO_2906_Ext_rev	gacaccgagtcttcgacctc	
HVO_1594_Int_Fwd	gcatcctggaactcgaagac	Internal checks for *HVO_1594*
HVO_1594_Int_Rev	aggtgcaggtggagtagacg	
HVO_1594_Ext_Fwd	ttcagcgaaccagacgctac	External checks for *HVO_1594*
HVO_1594_Ext_Rev	cggtctttgatttgggactg	
HVO_1475_int_Fwd	aactccttgctcccttcgtt	Internal

HVO_1475_int_Rev	gtctcgctcgaacagtaccc	checks for *HVO_1475*
HVO_1475_Ext_fwd	gacgtgacagtcgaggtggt	External checks for *HVO_1475*
HVO_1475_Ext_rev	agtacgcgtggagccactac	
HVO_0574_Int_Fwd	agttcccgctggagacagt	Internal checks for *HVO_0574*
HVO_0574_Int_Rev	cgaagtcgagtcgttcgtc	
HVO_0574_Ext_fwd	agtccgagcctcgaagagtt	External checks for *HVO_0574*
HVO_0574_Ext_rev	aggacagtcgcaagtccagt	
HVO_1631_Int_Fwd	aggacctgtccctcgtaggt	Internal checks for *HVO_1631*
HVO_1631_Int_Rev	aacagatcgtcgaagccatc	
HVO_1631_Ext_Fwd	gctacctgtgccctgaatgt	External checks for *HVO_1631*
HVO_1631_Ext_Rev	gggaactcctgtgcgataga	
HVO_0916_Int_Fwd	ctcccagtcgaccttgatgt	Internal checks for *HVO_0916*
HVO_0916_Int_Rev	tcgcggagttctacacgag	
HVO_0916_Ext_Fwd	cgtccacaagagctacatcg	External checks for *HVO_0916*
HVO_0916_Ext_Rev	gggtaatctcacgcaatggt	

HVO_2744_Int_Fwd	tcgtagccggtgaagctact	Internal checks for *HVO_2744*
HVO_2744_Int_Rev	gggatgaacacctcggtcta	
HVO_2744_Ext_Fwd	gtcttcgaccagcgtgttct	External checks for *HVO_2744*
HVO_2744_Ext_Rev	gtgcgcccgtggatagac	
HvO_0253_Ext_F	cgggaccggaattaagagtt	External checks for *HVO_0253*
HvO_0253_Ext_R	tgccatgtcagtaagccaag	
HvO_0253_Int_F	aacgcgtcttcgacatcaa	Internal checks for *HVO_0253*
HvO_0253_Int_R	gtcgtcgtgctcgtctaacc	
HVO_1979_Ext_Fwd	cgacgattctcgaagctgat	External checks for *HVO_1979*
HVO_1979_Ext_Rev	gtcgagcaggacgaagtagg	
HVO_1979_Int_Fwd	gggtgtactgctcgatggtc	Internal checks for *HVO_1979*
HVO_1979_Int_Rev	gtgcagttcctcctcgacat	
HVO_2747_Int_Fwd	aaaagtcccgttccgctact	Internal checks for *HVO_2747*
HVO_2747_Int_Rev	acgaaatcctcaacgtcgtc	
HVO_2747_Ext_Fwd	gcgaggttcgattcgaggtc	External checks for
HVO_2747_Ext_Rev	tcgaggcgaggcggaagagc	

		HVO_2747
HVO_1717_Ext_Fwd	tcgcgcaccgcaatgagctt	External checks for *HVO_1717*
HVO_1717_Ext_Rev	aggttctggaacgcctgcaa	
HVO_1717_Int_Fwd<br>HVO_1717_Int_Rev	acgagctgttcgcgtcgctt<br>acacgtcgctgtcgcggatt	Internal checks for *HVO_1717*
HVO_1716_Ext_Fwd	gccgtcgtgctcgaagagaa	External checks for *HVO_1716*
HVO_1716_Ext_Rev	ggcgactctgctcacctact	
HVO_1717_Int_Fwd	acgaactctacgcgctccac	Internal checks for *HVO_1716*
HVO_1717_Int_Rev	ggcgactctgctcacctact	
HVO_0929_Int_Fwd	tgctcgtcggcgactcgacc	External checks for *HVO_0929*
HVO_0929_Int_Rev	tcgcgaacgtcgccgtgaat	
HVO_0929_Ext_Fwd	gaataaaaccgaacccggtcgc	External checks for *HVO_0929*
HVO_0929_Ext_Rev	ggcatcgcgaaggaaatcaac	
pJAM202c cloning		
HVO_0253_NdeI_Fwd	atgccatatgatgagcgacgacgtaca	Cloning of *HVO_0253* into pJAM202
HVO_0253_BlpI_Rev	atgcgctgagttacgcgtcgtcgtcgtgct	

Hvo_2747_ndei_fwd	atgccatatgatgacacgtacggagagcgg	Cloning *HVO_2747* into pJAM
Hvo_2747_blpi_rev	atgcgctgagcctgtccgtcgcgtccgacga	
HVO_0339_NdeI_Fwd	atgccatatgatgaccgtcatcggcctcga	Cloning of *HVO_0339* into pJAM202
HVO_0339_BlpI_Rev	atgcgctgagtcagcgctccgggtgcgt	
Hvo697_fwd	atgccatatgatggacgacatcgaaatcga	Cloning of *HVO_0697* into pJAM202
Hvo697_rev	atgcgctgagtcagccgatgtaccggaggt	
HVO_1979_NdeI_Fwd	atgccatatgatgccatatgatgagcatccttgagg	Cloning of *HVO_1979* into pJAM202
HVO_1979_BlpI_Rev	atgcgctgagatgcgctcagctcagtccttgaagaa	
HVO_2001_Fwd	gctcagctacgtcgggtgaacctaaga	Cloning of *HVO_2001* into pJAM202
HVO_2001_Rev	catatgcgcgaccacttcgaactc	

Supplemental Table 4 Salt and temperature tolerance of tested deletion mutants

26˚C

**Strain**	**12%**	**14%**	**16%**	**18%**	**23%**	**25%**
**H26**	+	+	+	+	**-**	**-**
HVO_0916	**-**	**-**	**-**	**-**	**-**	**-**
HVO_1631	**-**	**-**	**-**	**-**	**-**	**-**
HVO_2477	+	++	++	++	**-**	**-**
HVO_2493	+	+	+	+	**-**	**-**
HVO_1105	+	+	+	+	**-**	**-**
HVO_1105 ***HVO_2008***	+	+	+	+	**-**	**-**
HVO_2001	**-**	**-**	**-**	**-**	**-**	**-**
HVO_2008	+	+	+	+	**-**	**-**
HVO_1717	+	+	+	+	**-**	**-**
HVO_1716	+	+	+	+	**-**	**-**
HVO_0253	+	+	+	+	**-**	**-**
HVO_1803	+	+	+	+	**-**	**-**
HVO_B0354	+	+	+	+	**-**	**-**
HVO_1594	+	+	+	+	**-**	**-**
HVO_0390	+	+	+	+	**-**	**-**
HVO_2888	+	+	+	+	**-**	**-**
HVO_1378	+	+	+	+	**-**	**-**
HVO_1852	+	+	+	+	**-**	**-**

HVO_0658	+	+	+	+	-	-
HVO_2478	+	+	+	+	-	-
HVO_2736	+	+	+	+	-	-
HVO_1475	+	+	+	+	-	-
HVO_0236	+	+	+	+	-	-

30˚C

**Strain**	**12%**	**14%**	**16%**	**18%**	**23%**	**25%**
**H26**	++	++	++	++	-	-
HVO_0916	-	-	-	-	-	-
HVO_1631	++	++	++	++	-	-
HVO_2477	++	++	++	++	-	-
HVO_2493	++	++	++	++	-	-
HVO_1105	++	++	++	++	-	-
HVO_1105 ***HVO_2008***	++	++	++	+	-	-
HVO_2001	++	++	++	++	-	-
HVO_2008	++	++	++	++	-	-
HVO_1717	++	++	++	++	-	-
HVO_1716	++	++	++	++	-	-
HVO_0253	++	++	++	++	-	-
HVO_1803	++	++	++	++	-	-
HVO_B0354	++	++	++	++	-	-

HVO_1594	++	++	++	++	-	-
HVO_0390	++	++	++	++	-	-
HVO_2888	++	++	++	++	-	-
HVO_1378	++	++	++	++	-	-
HVO_1852	++	++	++	++	-	-
HVO_0658	++	++	++	++	-	-
HVO_2478	++	++	++	++	-	-
HVO_2736	++	++	++	++	-	-
HVO_1475	++	++	++	++	-	-
HVO_0236	++	++	++	++	-	-

37˚C

**Strain**	**12%**	**14%**	**16%**	**18%**	**23%**	**25%**
**H26**	++	++	++	++	-	-
HVO_0916	-	-	-	-	-	-
HVO_1631	++	++	++	++	-	-
HVO_2477	++	++	++	++	-	-
HVO_2493	++	++	++	++	-	-
HVO_1105	++	++	++	++	-	-
HVO_1105 ***HVO_2008***	++	++	++	+	-	-
HVO_2001	++	++	++	++	-	-
HVO_2008	++	++	++	++	-	-

HVO_1717	++	++	++	++	-	-
HVO_1716	++	++	++	++	-	-
HVO_0253	++	++	++	++	-	-
HVO_1803	++	++	++	++	-	-
HVO_B0354	++	++	++	++	-	-
HVO_1594	++	++	++	++	-	-
HVO_0390	++	++	++	++	-	-
HVO_2888	++	++	++	++	-	-
HVO_1378	++	++	++	++	-	-
HVO_1852	++	++	++	++	-	-
HVO_0658	++	++	++	++	-	-
HVO_2478	++	++	++	++	-	-
HVO_2736	++	++	++	++	-	-
HVO_1475	++	++	++	++	-	-
HVO_0236	++	++	++	++	-	-

44˚C

**Strain**	**12%**	**14%**	**16%**	**18%**	**23%**	**25%**
**H26**	++	++	++	++	++	+
HVO_0916	+	+	++	++	+	-
HVO_1631	++	++	++	++	++	-
HVO_2477	++	++	++	++	++	+
HVO_2493	++	++	++	++	++	+

HVO_1105	++	++	++	++	++	+
HVO_1105 ***HVO_2008***	++	++	++	++	++	+
HVO_2001	++	++	++	++	++	+
HVO_2008	++	++	++	++	++	+
HVO_1717	++	++	++	++	++	+
HVO_1716	++	++	++	++	++	+
HVO_0253	++	++	++	++	++	+
HVO_1803	++	++	++	++	++	+
HVO_B0354	++	++	++	++	++	+
HVO_1594	++	++	++	++	++	+
HVO_0390	++	++	++	++	++	+
HVO_2888	++	++	++	++	++	+
HVO_1378	++	++	++	++	++	+
HVO_1852	++	++	++	++	++	+
HVO_0658	++	++	++	++	++	+
HVO_2478	++	++	++	++	++	+
HVO_2736	++	++	++	++	++	+
HVO_1475	++	++	++	++	++	+
HVO_0236	++	++	++	++	++	+

50˚C

**Strain**	**12%**	**14%**	**16%**	**18%**	**23%**	**25%**

**H26**	**-**	**-**	**-**	++	++	++
HVO_0916	**-**	**-**	**-**	+	+	+
HVO_1631	**-**	**-**	**-**	++	++	++
HVO_2477	**-**	**-**	**-**	++	++	++
HVO_2493	**-**	**-**	**-**	++	++	++
HVO_1105	**-**	**-**	**-**	++	++	++
HVO_1105 ***HVO_2008***	**-**	**-**	**-**	++	++	++
HVO_2001	**-**	**-**	**-**	++	++	++
HVO_2008	**-**	**-**	**-**	++	++	++
HVO_1717	**-**	**-**	**-**	++	++	++
HVO_1716	**-**	**-**	**-**	++	++	++
HVO_0253	**-**	**-**	**-**	++	++	++
HVO_1803	**-**	**-**	**-**	++	++	++
HVO_B0354	**-**	**-**	**-**	++	++	++
HVO_1594	**-**	**-**	**-**	++	++	++
HVO_0390	**-**	**-**	**-**	++	++	++
HVO_2888	**-**	**-**	**-**	++	++	++
HVO_1378	**-**	**-**	**-**	++	++	++
HVO_1852	**-**	**-**	**-**	++	++	++
HVO_0658	**-**	**-**	**-**	++	++	++
HVO_2478	**-**	**-**	**-**	++	++	++
HVO_2736	**-**	**-**	**-**	++	++	++

HVO_1475	**-**	**-**	**-**	++	++	++
HVO_0236	**-**	**-**	**-**	++	++	++

References

1. **Allers, T., H. Ngo, M. Mevarech, and R. Lloyd.** 2004. Development of additional selectable markers for the halophilic archaeon *Haloferax volcanii* based on the *leuB* and *trpA* genes. *Appl Environ Microbiol* **70:**943-53.
2. **El Yacoubi, B., G. Phillips, I. Blaby, C. Haas, Y. Cruz, J. Greenberg, and V. de Crécy-Lagard.** 2009. A Gateway platform for functional genomics in *Haloferax volcanii*: deletion of three tRNA modification genes. *Archaea* **2:**211-9.
3. **Kaczowka, S., and J. Maupin-Furlow.** 2003. Subunit topology of two 20S proteasomes from *Haloferax volcanii. J Bacteriol* **185:**165-74.
4. **Phillips, G., V. Chikwana, A. Maxwell, B. El-Yacoubi, M. Swairjo, D. Iwata-Reuyl, and V. de Crécy-Lagard.** 2010. Discovery and characterization of an amidinotransferase involved in the modification of archaeal tRNA. *J Biol Chem* **285:**12706-13.
5. **Wendoloski, D., C. Ferrer, and M. Dyall-Smith.** 2001. A new simvastatin (mevinolin)-resistance marker from *Haloarcula hispanica* and a new *Haloferax volcanii* strain cured of plasmid pHV2. *Microbiology* **147:**959-64.
